# Right-to-left-shunts in patients scheduled for neurosurgical intervention in semi-sitting position – a literature review based on two case scenarios

**DOI:** 10.1186/s12871-024-02757-6

**Published:** 2024-10-16

**Authors:** Marina Nikolic, C. Eisner, J. O. Neumann, D. Haux, S. M. Krieg, M. O. Wielpütz, M. A. Weigand, U. Tochtermann, Dania Fischer

**Affiliations:** 1 Medical Faculty Heidelberg, Department of Anesthesiology, Heidelberg University, Heidelberg, Germany; 2 Medical Faculty Heidelberg, Department of Neurosurgery, Heidelberg University, Heidelberg, Germany; 3 Medical Faculty, Department of Diagnostic and Interventional Radiology, Heidelberg University, Heidelberg, Germany; 4 Medical Faculty Heidelberg, Department of Cardiothoracic Surgery, Heidelberg University, Heidelberg, Germany

**Keywords:** Right-to-left-shunt, Semi-sitting position, Perioperative TEE, Patent foramen ovale

## Abstract

**Background:**

Neurosurgery performed in the semi-sitting position provides advantages for certain procedures. However, this approach is associated with potential complications, particularly venous air embolism. Due to typically negative venous pressure at the wound site, air can be drawn into the veins. This risk is especially high in patients presenting with an intra- or extracardiac right-to-left-shunt. Transoesophageal echocardiography can be used to detect a patent foramen ovale or other possible pulmonary-systemic shunt before placing the patient in the sitting position.

**Case presentation:**

In this report, we present two young patients undergoing scheduled microsurgical vestibular schwannoma removal in a semi-sitting position who were diagnosed with congenital heart defects during routine perioperative assessment to detect possible intracardiac right-to-left shunts, using pre- and intraoperative transesophageal echocardiography (TEE) and additionally conducting an agitated saline bubble study under Valsalva manoeuvre. Patient A was diagnosed with a persistent left superior vena cava and Patient B with an unroofed coronary sinus (UCS). These findings confronted the anesthesiological and surgical teams with difficult individual decisions regarding further perioperative management.

**Conclusions:**

Perioperative transesophageal echocardiography is a diagnostic tool to both detect intraoperative position-related air embolisms and to rule out intracardiac right-to-left shunts, e.g. a patent foramen ovale, in order to decide for or against a (semi-)sitting position. Depending on the surgical circumstances a semi-sitting positioning of patients presenting with an intracardiac right-to-left-shunt, e.g. a PFO, can be feasible in individual cases if there is an implemented therapeutic algorithm to immediately terminate significant venous air entry. However, since certain other intra- or extracardiac right-to-left-shunts, such as here presented PLSVC or UCS, are rare, there is no definitive way of estimating the amount of entered air through detected shunts or anomalous vessels. Therefore, it is recommended to avoid a (semi-)sitting position in favour of a lateral or prone position for a patient undergoing intracranial surgery, once the perioperative TEE shows air bubbles in the left atrium or ventricle whose origins cannot be defined solely through TEE for certain in order to ensure patient safety by minimizing the risk of intraoperative paradoxical air embolisms.

## Background

Certain neurosurgical procedures, particularly tumor resections in the posterior fossa, require the patient to be in a semi-sitting position in order to facilitate the surgical approach [1, 2]. Despite its several surgical advantages, the semi-sitting patient positioning carries a relevant risk of venous air entry possibly leading to paradoxical venous air embolism (VAE). A patent foramen ovale (PFO) with right-to-left-shunting has been discussed abundantly as a possible contraindication for this type of patient positioning whereupon the associated incidence of paradoxical venous air embolism to the heart, lungs or brain has been reported to be between 0% and 14% in the literature [3]. 

**Fig. 1:**
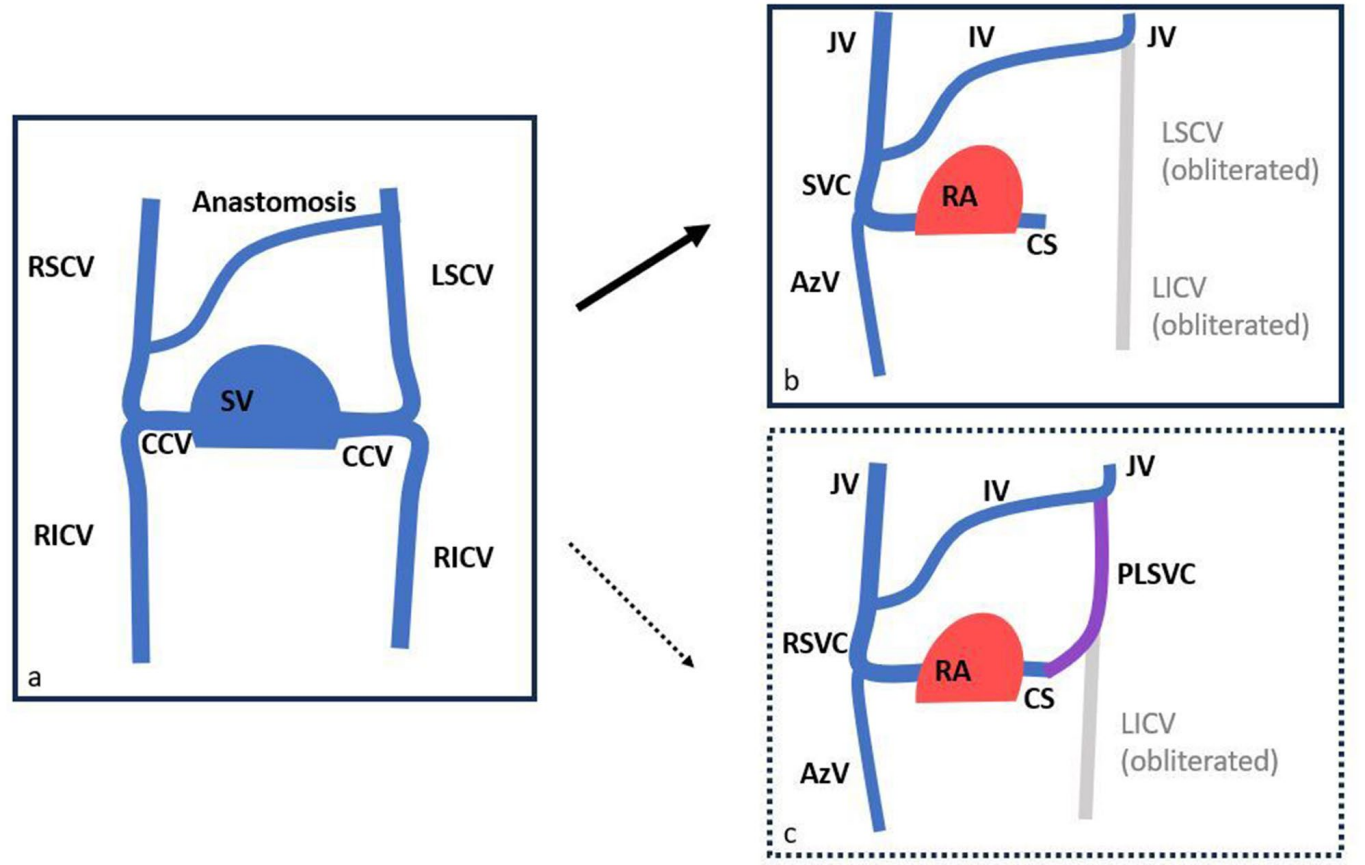
Embryologic developmental stages of the primitive venous system (simplified and adapted from Azizova et al.) [4]. **a**) the double horned sinus venosus (SV) is yet a separate part of the human heart in the 4th week of intrauterine development. The right and left superior and inferior cardinal veins (RSCV, LSCV, RICV, LICV) drain into the SV via the common cardinal veins (CCV). The RSCV and the LSCV are connected via an anastomosis. **b**) up to the 12th week the RSCV and the LSCV form the jugluar veins (JV) and the innominate vein (IV). The right CCV and part of the RSCV form the superior vena cava (SVC). The azygos vein (AzV) is formed by the RICV, and the LICV obliterates. The right horn of the SV becomes part of the right atrium (RA) whereas the left horn forms the coronary sinus (CS). **c**) if obliteration of the LSCV does not occur, the persistens left superior vena cava (PLSVC) remains. It can drain into the left atrium directly or indirectly through the CS or, as seen with Patient A, via pulmonary veins. The SVC on the right side is then labeled the right superior vena cava (RSVC)

Since a PFO is not the only condition that can result in a right-to-left shunt, we report on two rare cases of cardiac shunts (Patients A and B), who were scheduled for microsurgical vestibular schwannoma resection in a semi-sitting position. During routine perioperative transesophageal echocardiography, Patient A was found to have a previously undiagnosed extracardiac right-to-left shunt, which was identified via CT scan as a persistent left superior vena cava (PLSVC). PLSVC is a rare and mostly incidental finding in clinical practice, with a prevalence ranging from about 0.2–3% in the general population, although it is one of the most common thoracic venous anomalies (Fig. 1) [4]. The majority of PLSVC cases drain into the right atrium via the coronary sinus. Less commonly, it drains into the left atrium, either directly or indirectly through the left pulmonary veins [4]. Patients with PLSVC are typically asymptomatic, however, this anomaly can complicate various therapeutic interventions, such as central venous catheterization and surgical procedures [5]. 

**Fig. 2:**
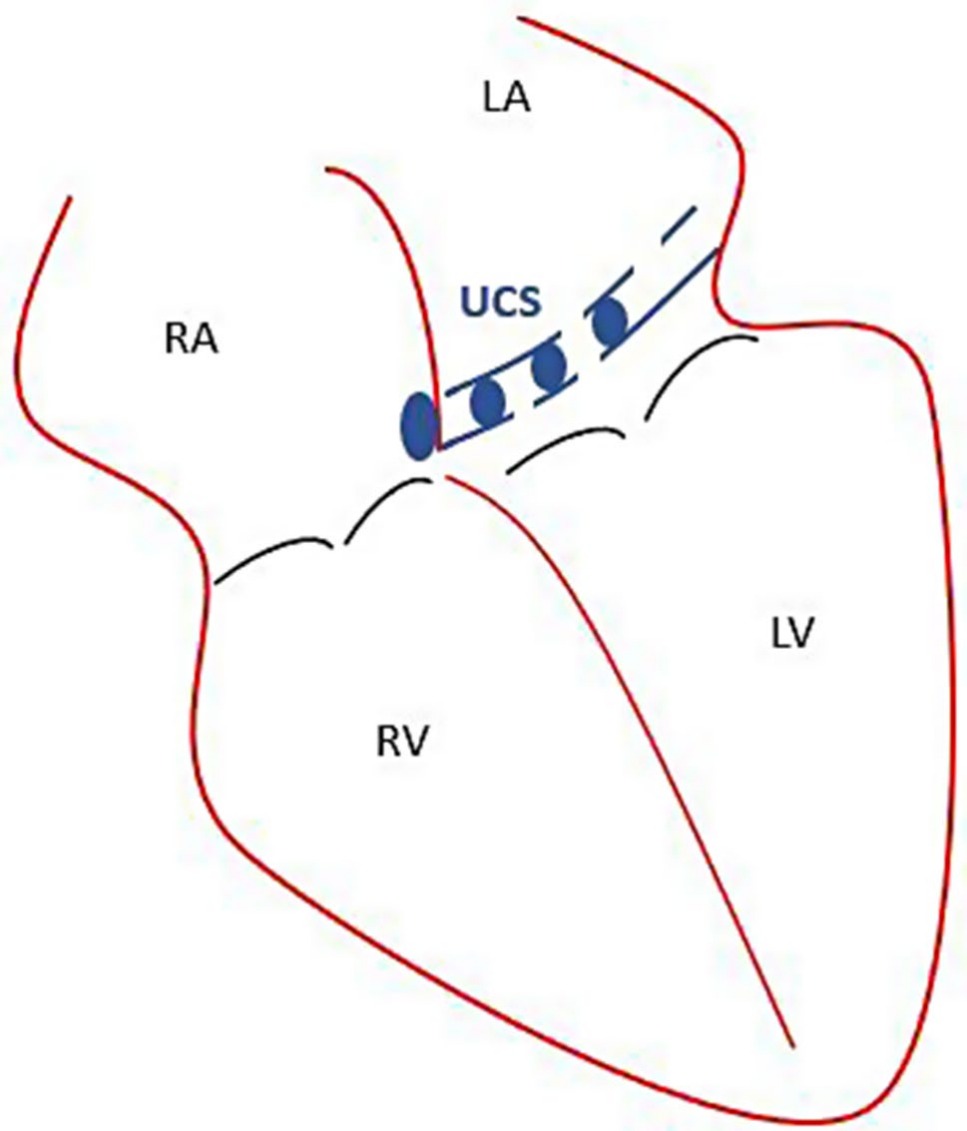
Schematic drawing of an unroofed coronary sinus (UCS). LA: left atrium; RA: right atrium; LV: left ventricle; RV: right ventricle. (adapted from Pérez Matos et al.) [11]

Another congenital heart defect (CHD) with an even lower incidence, which can also result in a right-to-left shunt, is the unroofed coronary sinus (UCS) (Fig. 2). This rare form of atrial septal defect has been primarily documented in case reports [6–9]. Kirklin and Barratt-Boyes described different types of UCS in the 1980s, distinguishing between completely versus partially unroofed conditions and those associated with or without a persistent left superior vena cava (PLSVC) [10]. One of the cases presented in this report is a young woman, who was scheduled for neurosurgical vestibular schwannoma removal in a semi-sitting position. Perioperative transesophageal echocardiography (TEE) revealed a suspected UCS, leading to a decision against the semi-sitting position in favor of a lateral approach to avoid the potentially catastrophic event of a paradoxical VAE.

## Case presentation

### Patient A

A 34-year-old male patient was scheduled for neurosurgical removal of a left-sided vestibular schwannoma in a semi-sitting position. Apart from a diagnosis of multiple sclerosis in 2018, there were no other preexisting conditions in the patient’s history. The left-sided schwannoma was incidentally discovered during regular MRI follow-up examinations for multiple sclerosis. Although the patient was mostly asymptomatic, with only mild hypacusis, surgical removal was recommended due to the tumor’s persistent growth, and the patient was referred to our hospital.

**Fig. 3:**
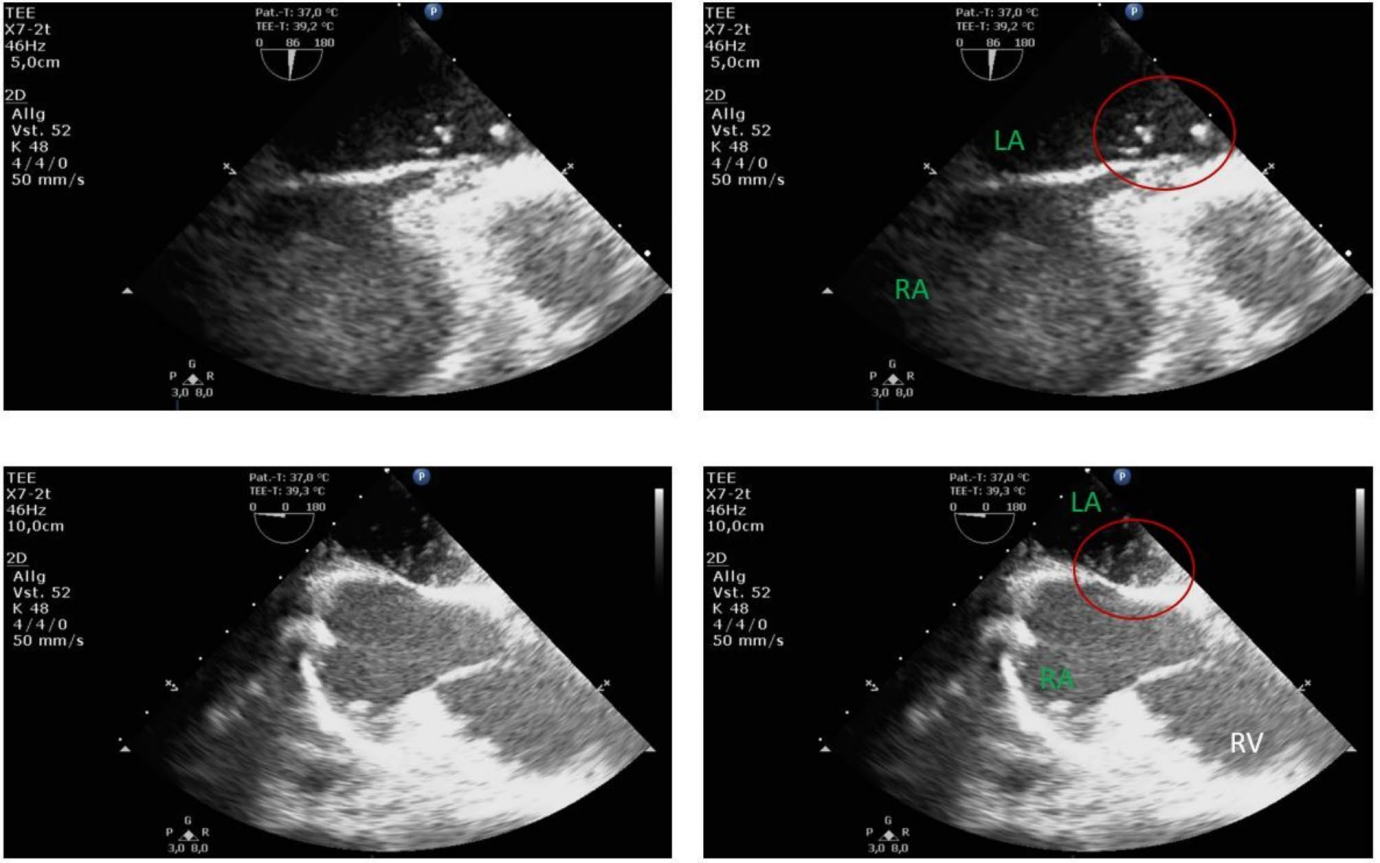
Patient A’s perioperative side-by-side images of the echocardiographic assessment of possible cardiac right-to-left shunts using bubble study showing air bubbles (circled, red) solely in the left atrium (LA) leaving the right atrium (RA) bubble free. upper two images: mid esophageal modified bicaval view at 86°. lower two images: mid esophageal two chamber view at 0°, focused to the right heart. LA: left atrium; RA: right atrium; RV: right ventricle; red circle: air bubbles

General anesthesia was administered without complications prior to surgery. Following our standardized approach, the patient was equipped with an endotracheal tube, an arterial cannula, a urinary catheter, and a central venous catheter. The catheterization of the left inferior jugular vein was performed under ultrasound guidance. The placement of the left central venous catheter was easy, without resistance or unusual complications. To detect a patent PFO as a potential right-to-left shunt, the interatrial septum was examined using contrast echocardiography (microbubbles) and color Doppler. This assessment was conducted during the release of a Valsalva maneuver, as this is when right-to-left flow is most likely to occur. This study revealed a significant number of bubbles solely in the left atrium, leaving the right atrium bubble-free (Fig. 3).

**Fig. 4:**
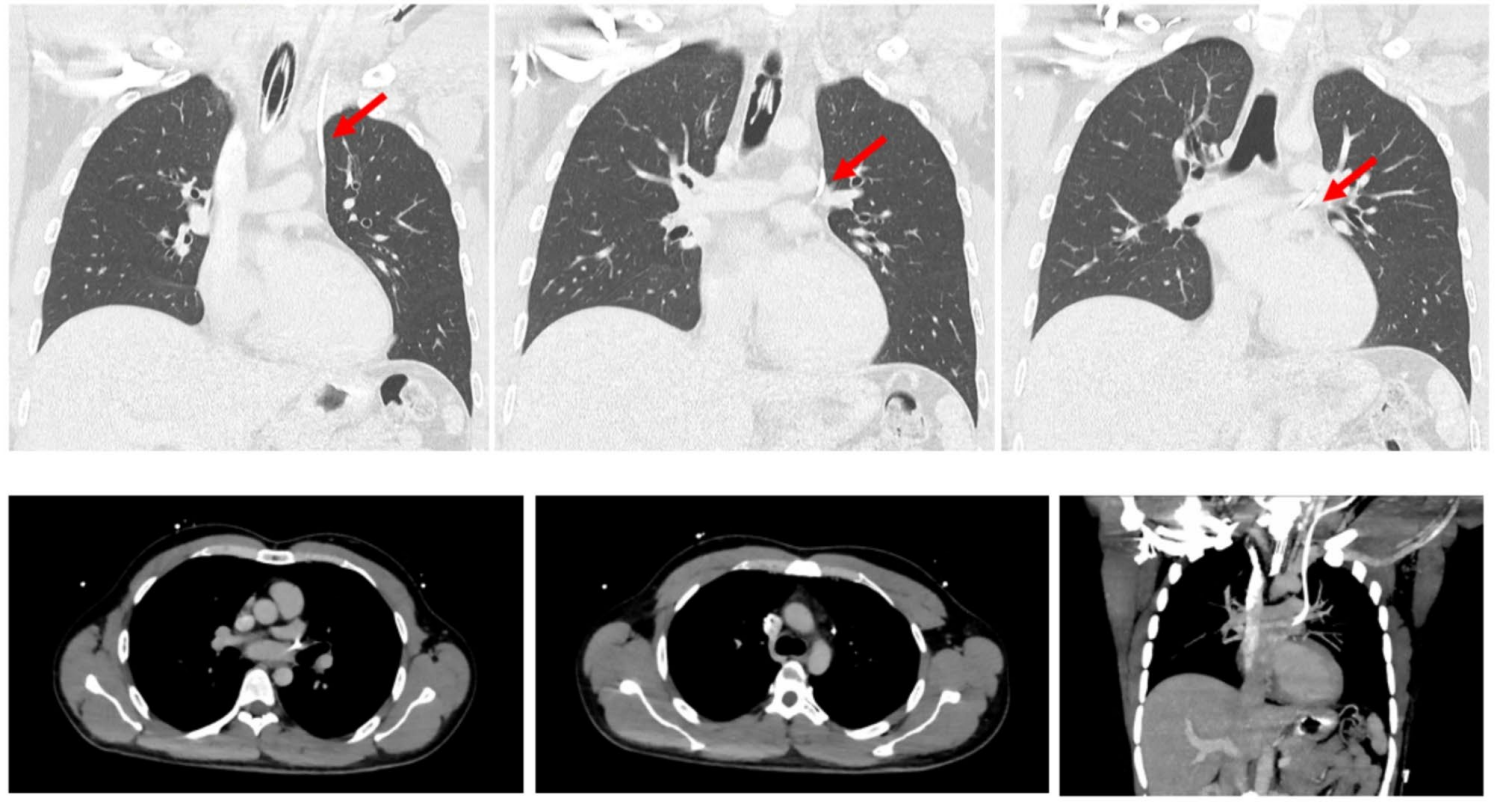
Position of the left sided central line with drainage into the left upper pulmonary vein shown in patient A’s CT scan (both lung window and maximum intensity projection)

Manometry monitoring showed a venous pressure curve, but blood gas analysis from the left central line appeared to be arterial measurements, suggesting its possible localization in a pulmonary vein (Table 1). Consequently, the left central venous catheter was not used for administering medications, and another catheter was placed into the right internal jugular vein under ultrasound guidance. A CT scan of the thorax was initially interpreted to show an incorrect mediastinal position of the left central line with possible perforation of both the left brachiocephalic vein and the left upper pulmonary vein. However, further investigation of the CT images involved an interdisciplinary approach with radiologists, cardiac surgeons, neurosurgeons, and anesthesiologists. After careful analysis and discussion, the central line was identified to be inside a filiform persistent left superior vena cava (Fig. 4).

**Table 1:** Patient A’s blood gas analysis with sample taken from the left sided central line with a high pO2 indicating arterial blood (in bold)

Time	15:33	17:40		
pH	7.41	7.40		(7.37–7.45)
pO₂	**294**	230	mmHg	(35–45)
base excess	-1.2	-1.0	mmol/l	(-2 - +3)
K^+^	3.84	4.23	mmol/l	(3.6–4.8)
Na^+^	137	139	mmol/l	(135–145)
Ca^2+^	1.19	1.22	mmol/l	(1.15–1.35)
Cl^−^	106	108	mmol/l	(95–105)
COHb	0.2	0.1	%	(< 2.0)
MetHb	0.5	0.4	%	(< 1.5)
Hb	13.1	13.8	g/dl	(12–15)
Hct	39	41	%	
Glc	84	100	mg/dl	(65–100)
SO₂	99.5	99.1	%	
source	**venous**	arterial		

**Fig. 5:**
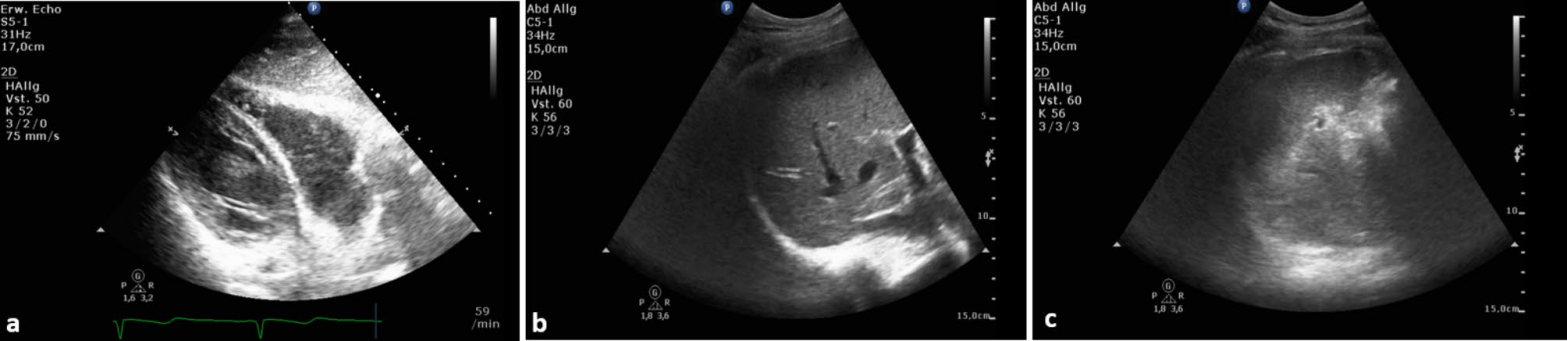
Postoperative exclusion of **a**) cardiac tamponade, **b**) hematothorax - right pleural cavity) and **c**) left pleural cavity of patient A

The microsurgical removal of the vestibular schwannoma was successfully completed in a modified semi-sitting positioning of the patient with a mild elevation of the head and the upper body. A thorough and continuous TEE monitoring was performed throughout the whole procedure to ensure the early detection of possible air bubble entry to the heart. In the ICU, the left central line was removed without complications, followed by frequent echocardiographic exams to rule out cardiac tamponade and hemothorax (Fig. 5). The patient was extubated the following day and transferred to the general ward. He experienced a mild aggravation of his preexisting hypacusis, and initial postoperative vertigo was successfully treated with physical therapy. The patient was discharged after seven days.

### Patient B

Since the aforementioned neurosurgical procedure is frequently performed at our institution, a similar case occurred just a few weeks later. A 40-year-old female patient was found to have a previously unknown intracardiac right-to-left shunt during perioperative transesophageal echocardiography (TEE). She presented with mild numbness and paresthesia on the right side of her face, mild hypacusis with preserved useful hearing, and occasional unsteady gait. An MRI scan revealed a suspected neuroma of the vestibulocochlear and facial nerve, leading to the scheduling of neurosurgical removal of the mass in a semi-sitting position.

**Fig. 6:**
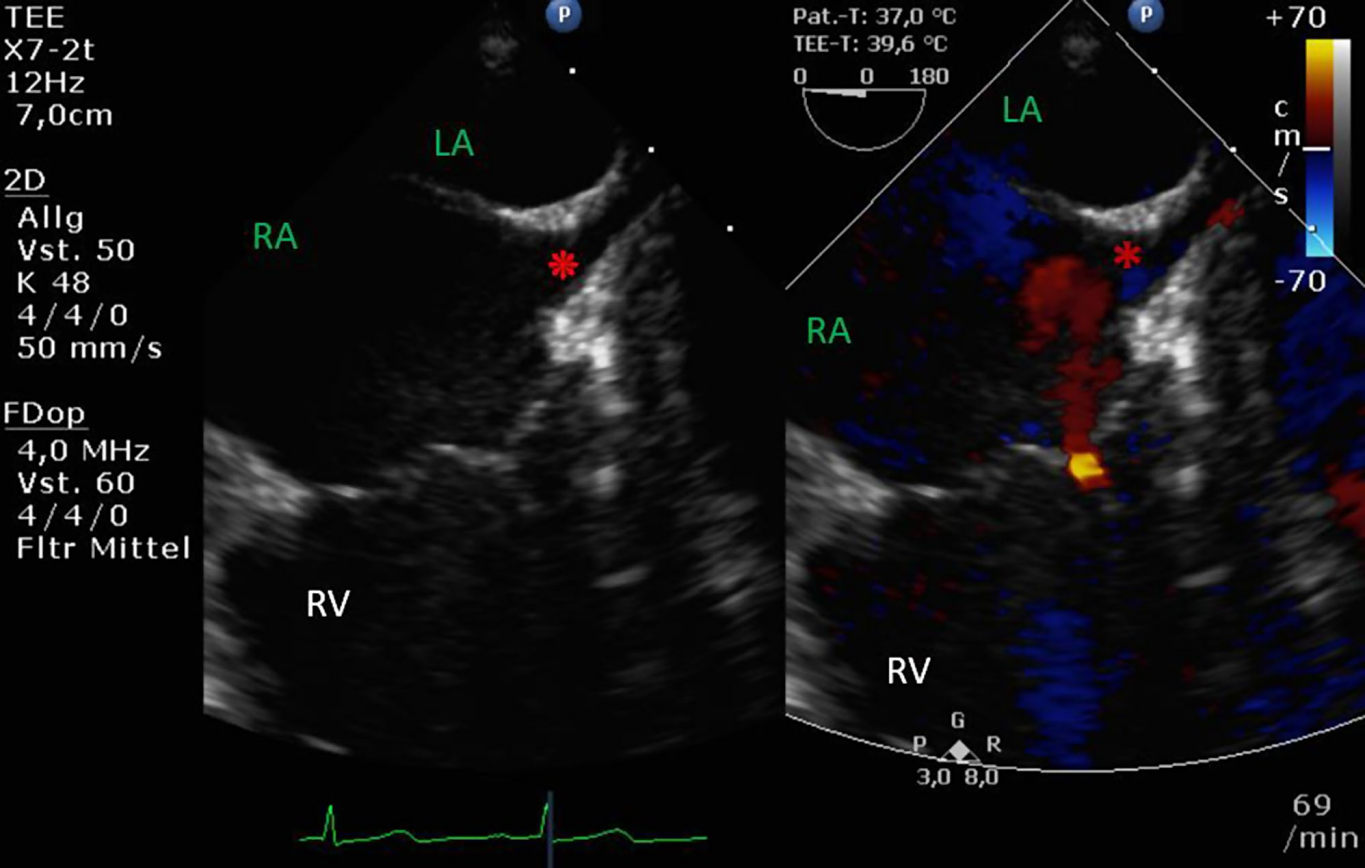
Perioperative comprehensive transesophageal echocardiographic exam of Patient B showing a suspected unroofed coronary sinus (UCS), mid esophageal two chamber view at 0°, focused to the right heart. LA: left atrium; RA: right atrium; RV: right ventricle; red circle: air bubbles; *: suspected unroofed coronary sinus

Standard anesthesiological management was conducted, including the induction of general anesthesia. The patient was equipped with an endotracheal tube, an arterial cannula for blood pressure monitoring, a urinary catheter, and a left-sided central venous line inserted under ultrasound guidance according to our standard operating procedure. Comprehensive transesophageal echocardiography was performed, and a bubble study with agitated saline was conducted to rule out possible right-to-left shunts. Air bubbles appeared immediately in the left atrium, followed by the left ventricle, and shortly thereafter in the right atrium. Further focused TEE views suggested an unroofed coronary sinus and the presence of a cor triatriatum dexter (CTD) (Fig. 6).

Similar to the investigative methods used for Patient A, blood gas analysis of the central line blood sample showed a high pO2 value (300mmHg), leading the anesthesiological team to assume the sample was taken from a pulmonary vein or the left atrium (Table 2). The left-sided central line was immediately removed and replaced with one inserted into the right jugular vein. Due to the inability to fully diagnose the underlying congenital heart defect (CHD) and anatomical variants using TEE alone, a recommendation was made against the semi-sitting position in favour of lateral positioning to avoid the risk of intraoperative VAE and potential stroke.

**Table 2:** Patient B’s blood gas analysis with sample taken from the left sided central line with a high pO2 indicating arterial blood (in bold)

Time	11:05	14:28		
pH	7.42	7.42		(7.37–7.45)
pCO₂	34.5	36.5	mmHg	(35–45)
pO₂	**300**	192	mmHg	
base excess	-2.1	-0.8	mmol/l	(-2 - +3)
K^+^	3.49	4.05	mmol/l	(3.6–4.8)
Na^+^	139	144	mmol/l	(135–145)
Ca^2+^	1.15	1.19	mmol/l	(1.15–1.35)
Cl^−^		112	mmol/l	(95–105)
COHb	0.3	0.3	%	(< 2.0)
MetHb	0.3	0.2	%	(< 1.5)
Hb	11.4	11.8	g/dl	(12–15)
Hct	34	35	%	
Glc	94	104	mg/dl	(65–100)
Lac	7.6	5.5	mg/dl	< 16
HCO₃^−^	21.9	23.3	mmol/l	
SO₂	99.5	98.9	%	
source	**venous**	arterial		

**Fig. 7:**
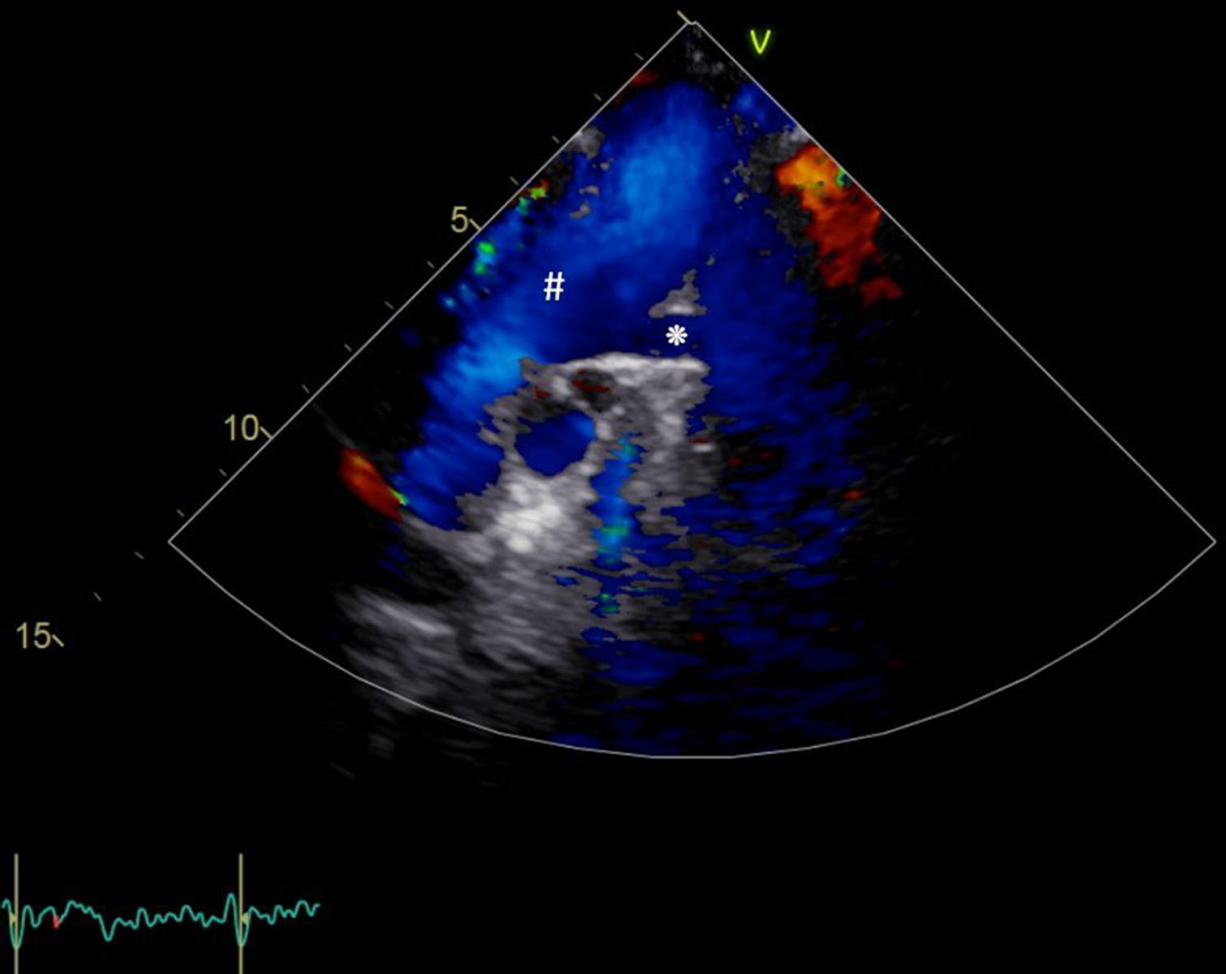
Patient B’s postoperative transthoracic echocardiography and vascular ultrasound exam showcasing a suspected anomalous pulmonary vein connection (APVC) (*) conducted by the department of pediatric cardiology and congenital heart diseases; #: innominate vein

The operation was successfully completed, and the patient was observed in the neurosurgical ICU for one night before being transferred to the general ward the following day. To further investigate the underlying CHD, a consultation was obtained from colleagues in the Department of Pediatric Cardiology and Congenital Heart Diseases. Transthoracic echocardiography and an additional repeated saline contrast study could not fully confirm the initially suspected unroofed coronary sinus or cor triatriatum dexter. However, the images indicated an anatomical variant of an approximately 8 mm wide vessel entering the innominate vein (Fig. 7), suggesting a pre-existing anomalous pulmonary venous connection (APVC). The patient was discharged five days after the operation with mild residual hypacusis.

## Discussion and conclusions

VAEs are known to be position-related complications during neurosurgical interventions, especially when the patient is in a sitting or semi-sitting position [12, 13]. To ensure intraoperative patient safety, various monitoring techniques, such as precordial Doppler ultrasound or TEE, can and should be used [14]. Besides detecting possible intracardiac air bubbles, pre- and intraoperative TEE, as commonly used in our institution for such procedures, can also identify an interatrial shunt, such as a PFO [15, 16]. Previous reviews have reported the incidence of VAE due to pre-existing PFO to range from 0 to 14% [3, 17]. The risk of this event can be significantly reduced by thorough perioperative screening for PFO in medical facilities with substantial experience in perioperative TEE and surgeries performed in the semi-sitting position. However, using only color Doppler to rule out intracardiac shunts might not be sufficient. Adding a contrast/ bubble study under Valsalva manoeuvre provides a more comprehensive assessment and could help detect congenital abnormalities that might otherwise be missed, which is crucial for minimizing the risk of venous air embolism.

In certain surgical scenarios, as described in Patient A’s case, lateral patient positioning may not always be feasible, leading to a case-by-case decision to accept the risk of VAE despite perioperative intra- or extracardiac shunt detection. In this particular case the decision was made upon an interdisciplinary discussion to enable a safe tumor removal under continuous periprocedural TEE monitoring. In such instances, therapeutic algorithms must be clearly defined to promptly terminate venous air entry and avoid catastrophic paradoxical VAE to the brain, heart, or lungs. Interestingly, as seen in our case scenarios, both patients presented with (extra)cardiac right-to-left shunts—an unknown PSLVC in Patient A and an unroofed coronary sinus in Patient B—which led to the detection of air bubbles in the left atrium immediately after agitated saline injection.

Given the extremely low prevalence of PSLVC and UCS, we do not recommend routine screening for these extracardiac congenital anomalies in all patients undergoing intracranial surgery in a sitting or semi-sitting position [4, 18]. However, if the underlying condition cannot be safely identified solely via perioperative TEE, we strongly recommend avoiding (semi-)sitting positions and switching to lateral patient positioning to prevent paradoxical VAE once air bubbles have been detected in the left atrium or ventricle during perioperative TEE. With the reported low incidences of rare congenital heart defects, it is impossible to determine the entered air volume or the risk of occluding essential blood vessels, which can lead to disastrous outcomes.

Additionally, the decision against (semi-)sitting positioning should apply to patients with previously diagnosed congenital heart defects with right-to-left shunts. We also suggest presenting rare and unusual findings to a broader group of specialists to collectively establish rare diagnoses, assess potential risks, and make appropriate therapeutic decisions to ensure patient safety.

## Data Availability

No datasets were generated or analysed during the current study.

## Abbreviations

APVC: anomalous pulmonary venous connection
AzV: azygos vein
ET: endotracheal tube
CCV: common cardinal vein
CHD: congenital heart defect
CS: coronary sinus
CT: computed tomography
CTD: cor triatriatum dexter
ICU: intensive care unit
IV: innominate vein
JV: jugular vein
LA: left atrium
LICV: left inferior cardinal vein
LSCV: left superior cardinal vein
LV: left ventricle
mm: millimetres
MRI: magnetic resonance imaging
PFO: patent foramen ovale
PLSVC: persistent left superior vena cava
RA: right atrium
RICV: right inferior cardinal vein
RSCV: right superior cardinal vein
RSVC: right superior vena cava
RV: right ventricle
SV: sinus venosus
TEE: transesophageal echocardiography
UCS: Unroofed coronary sinus
VAE: venous air embolism
